# Acute Posterior Multifocal Placoid Pigment Epitheliopathy (APMPPE): A Comprehensive Approach and Case Series: Systemic Corticosteroid Therapy Is Necessary in a Large Proportion of Cases

**DOI:** 10.3390/medicina58081070

**Published:** 2022-08-08

**Authors:** Ioannis Papasavvas, Alessandro Mantovani, Carl P. Herbort

**Affiliations:** 1 Retinal and Inflammatory Eye Diseases, Centre for Ophthalmic Specialized Care (COS), Rue Charles-Monnard 6, 1003 Lausanne, Switzerland; 2 Department of Ophthalmology, Valduce Hospital, 22100 Como, Italy

**Keywords:** primary inflammatory choriocapillaropathies (PICCPs), APMPPE/AMIC, indocyanine green angiography (ICGA), spectral domain optical coherence tomography (SD-OCT), enhanced depth imaging OCT (EDI-OCT), blue light fundus autofluorescence (BL-FAF)

## Abstract

*Background and objectives*: Acute posterior multifocal pigment epitheliopathy/acute multifocal ischaemic choriocapillaritis (APMPPE/AMIC) is part of the group of choriocapillaritis entities. The aim of this article was to report a series of patients with emphasis on the clinical presentation and treatment paradigms. *Materials and Methods*: Retrospective case series study performed in the Centre for Ophthalmic Specialised care (COS), Lausanne, Switzerland, on patients diagnosed from 2000 to 2021 with APMPPE/AMIC. Procedures performed at presentation and upon follow-up (when available) included best corrected visual acuity (BCVA), routine ocular examination, laser flare photometry (LFP) microperimetry (when available) and visual field testing. Imaging investigations included spectral domain optical coherence tomography (SD-OCT)/enhanced depth imaging OCT (EDI-OCT), OCT angiography (OCT-A) as well as fluorescein and indocyanine green angiography (FA, ICGA). The presence or not of prodromal systemic viral-like symptoms was noted. The localisation of lesions whether foveal or extrafoveal, divided the patients into 2 groups (foveal, peri-or parafoveal). Exclusion criteria were patients diagnosed with APMPPE/AMIC and a positive QuantiFERON test and/or VDRL-TPHA tests. *Results*: Nineteen (35 eyes) of 1664 new patients (1.14%) were diagnosed with APMPPE/AMIC and included in our study. 13 (68%) were male and 6 (32%) were female. The mean age was 33.1 ± 9.2 years. 16 (84%) patients mentioned a viral prodromal episode or other systemic symptoms, and 3 (16%) did not mention any episode before the onset of ocular symptoms. 15 (39%) out of 38 eyes had foveal localisation of the lesions, 20 (52.6%) had peri- or para-foveal localisations and 3 eyes were normal [3 unilateral cases (15%)]. Mean BCVA at presentation was 0.83 ± 0.24 for the whole group. It was 0.58 ± 0.28 for the group with foveal lesions, increasing to 0.97 ± 0.13 at last follow-up (*p* = 0.0028). For the group with extrafoveal lesions mean BCVA at presentation was 0.94 ± 0.18, improving to 1.18± 0.10 at last follow-up (*p* = 0.0039). 13 (68%) patients received prednisone treatment, of whom 2 (10%) received additionally at least one immunosuppressive agent, 4 (20%) patients received no treatment and in 2 patients the information was unavailable. All patients in the foveal lesion group received corticosteroid treatment except one who evolved to bilateral macular atrophy. *Conclusions*: APMPPE/AMIC is a primary choriocapillaritis. Although it is thought that the disease is self-limited, treatment is necessary in most cases, especially when lesions are located in the fovea.

## 1. Introduction

Acute posterior multifocal placoid pigment epitheliopathy (APMPPE) is a primary inflammatory choriocapillaropathy (PICCP) that was first described in 1968, by John Donald Macintyre Gass [1]. As its name indicates, the main lesion process was attributed to the retinal pigment epithelium (RPE). This pathophysiological explanation was, however, reoriented towards the preponderant role of inflammatory choriocapillaris hypo or non-perfusion at the origin of the disease process. Analysing fluorescein angiographic (FA) images showing early geographic hypofluorescence, Deutman came up with a more appropriate denomination of the disease by calling it Acute Multifocal Ischaemic Choriocapillaritis (AMIC) [2,3]. Indocyanine green angiography (ICGA) later showed that the FA hypofluorescent areas corresponded not only to a choriocapillaris perfusion delay but to non-perfusion throughout the angiographic sequence causing ischaemia to the outer retina-RPE complex [4].

**Figure 1:**
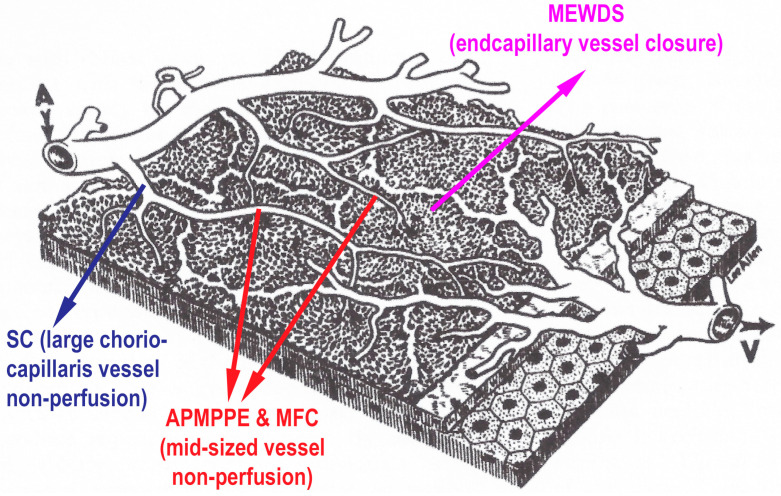
Clinicopathology of PICCPs according to vessel location of non-perfusion. Schematic drawing of the different levels of choriocapillaris vessel the occlusions of which determine the type of choriocapillaritis. (Reproduced from Papasavvas I, Herbort CP Jr. Diagnosis and Treatment of Primary Inflammatory Choriocapillaropathies (PICCPs): A Comprehensive Overview. Medicina (Kaunas). 2022 Jan 21;58(2):165. doi: 10.3390/medicina58020165. PMID: 35208488; PMCID: PMC8879059).

**Figure 2:**
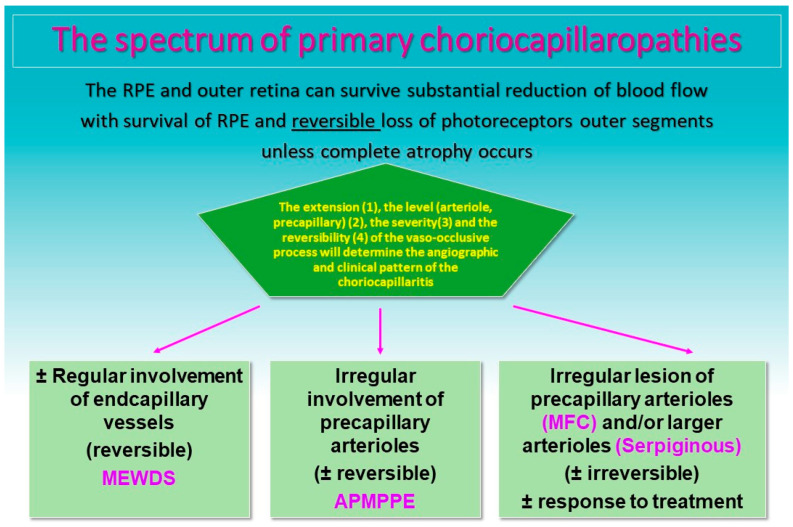
Clinicopathology of PICCPs according to the suspected type and extension of vessel non-perfusion. Non-perfusion of low-flow end capillary vessels causes the benign and reversible disease MEWDS. If larger choriocapillaris vessels or precapillary vessels are occluded the more severe diseases develop including APMPPE, MFC and SC [7]).

The common denominator of PICCPs is a dysfunction of the choriocapillaris perfusion of diverse severity and extension with grading from small end-capillary nonperfusion in Multiple Evanescent White Dot Syndrome (MEWDS) to progressively more proximal capillary involvement in Idiopathic Multifocal Choroiditis (MFC), APMPPE/AMIC and Serpiginous Choroiditis (SC) [5,6] (Figure 1 and Figure 2). It is called primary because there is no known cause at the origin of these entities.

In APMPPE/AMIC, inflammatory non-perfusion affects larger choriocapillaris or pre-choriocapillaris vessels (Figure 1), with more or less widespread involvement. Its particularity is the variability of involvement from limited non-perfused ICGA hypofluorescent areas to more severe and extensive lesions. It is therefore not possible to attribute a mean expected severity level of involvement to cases of this condition. Indeed, cases may resolve without treatment as it is usually reported in textbooks. It is, however, far from rare to have severe and extended ischaemic areas with potentially deleterious consequences, especially when the central macula is involved. In the latter cases, systemic corticosteroid therapy, with or without immunosuppressants, seems to be absolutely required [8,9,10,11]. APMPPE/AMIC develops preponderantly in young adults, being possibly slightly more frequent in men [12]. A viral or febrile episode or other systemic manifestation has been reported to precede the ocular disease in approximately half of the patients, an occurrence similar to other PICCPs. Sometimes the presumed virus can be tracked [13]. As in other PICCPs, vaccination can be the trigger at the origin of APMPPE/AMIC [14,15,16]. Bilaterality, concomitant or with a slight delay from one eye to the other, and limitation to a single episode determine the characteristic clinical pattern. In case of unilaterality and/or recurrences the diagnosis should be questioned and can be considered either as atypical APMPPE/AMIC or as a non-classifiable choriocapillaritis [5]. Mixed forms between APMPPE/AMIC and SC (Ampiginous choroiditis) can also occur and should be differentiated [17].

Symptoms include a decrease in visual acuity depending on the localisation of lesions in the macula, subjective scotomas and photopsias [12]. Anterior segment involvement, exceptionally including synechiae, is not a rare occurrence and subclinical anterior segment inflammation can be looked for with laser flare photometry [18].

**Figure 3:**
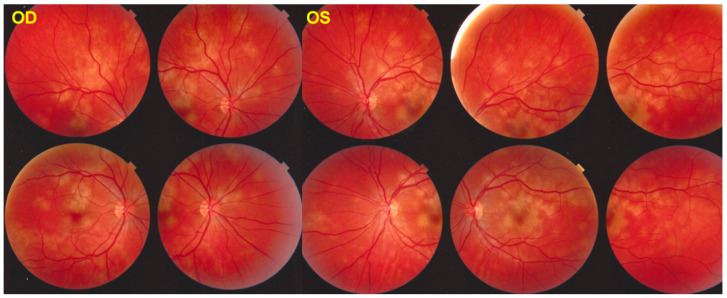
APMPPE/AMIC: Bilateral fundus pictures of a patient showing the characteristic placoid yellow lesions in the posterior pole and mid-periphery following a severe febrile episode with gastroenteritis.

The fundus appearance consists of the characteristic bilateral yellow-white discoloured “placoid” lesions in the posterior pole and mid-periphery after which the disease was originally named (Figure 3). Involvement may sometimes be at a slightly different stage in one and the contralateral eye [1,6]. In the differential diagnosis, infection-related conditions, such as acute syphilitic posterior placoid chorioretinitis (ASPPC) or tuberculosis-related serpiginoïd choroiditis (TB-SC), which can have similar presentations, should be considered [19,20].

Multimodal imaging contributes substantially to the diagnosis, the different imaging modalities pointing towards choriocapillaris non-perfusion and its consequences [5,21,22].

**Figure 4:**
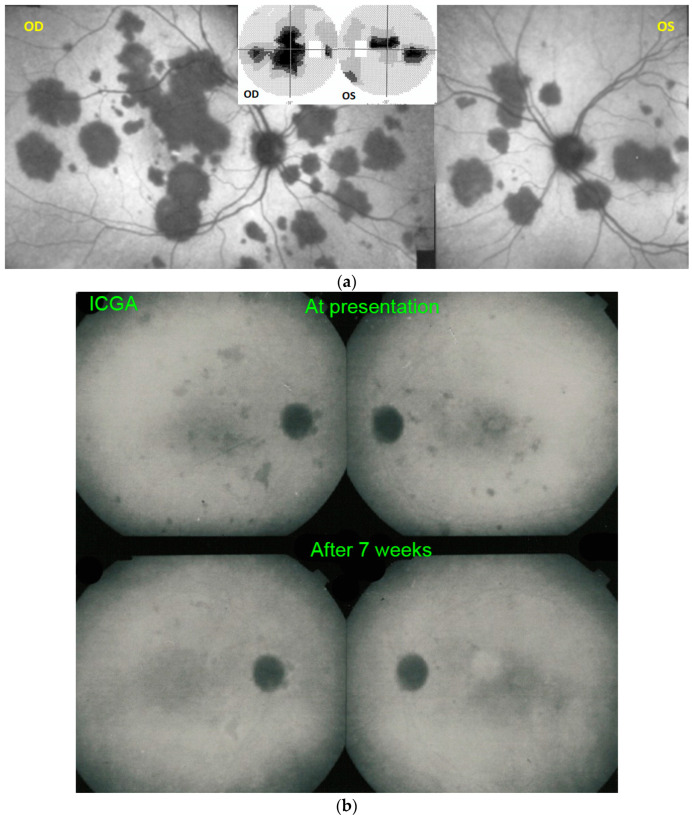
(**a**) APMPPE/AMIC: ICGA in a patient showing bilaterally scattered geographical partly confluent placoid hypofluorescent areas caused by choriocapillaris non-perfusion and corresponding visual field scotomas (inset). (**b**) APMPPE/AMIC: ICGA ODS in a case with limited areas of hypofluorescence (top two frames) that resolved without treatment after 7 weeks (bottom frames).

**Figure 5:**
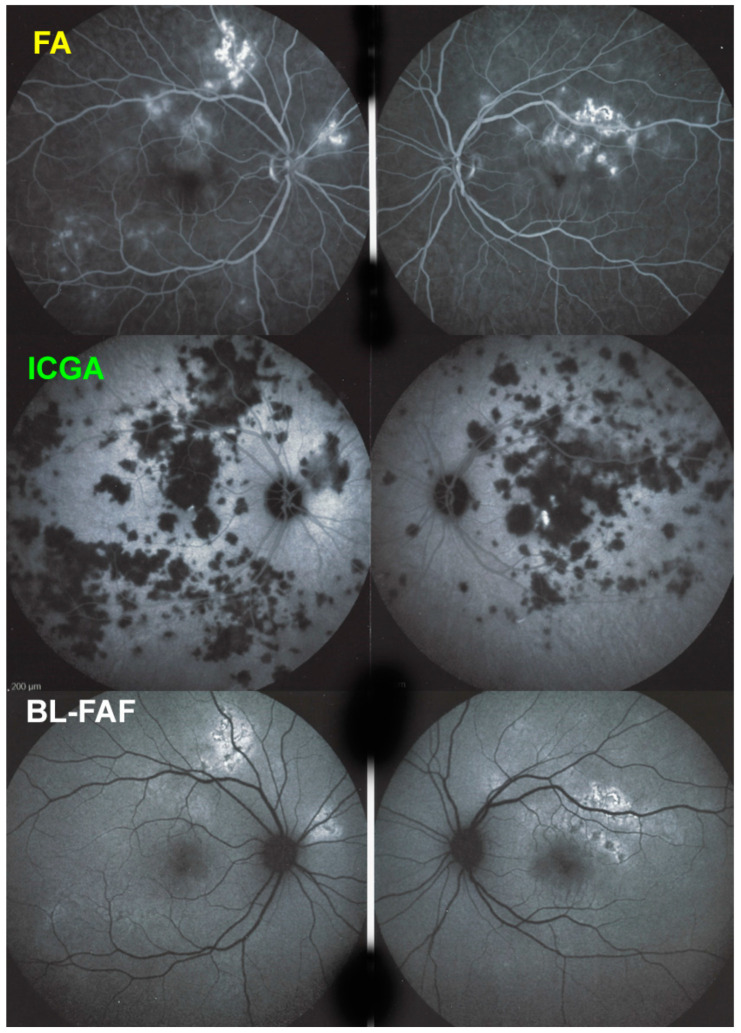
APMPPE/AMIC; FA & ICGA & BL-FAF at presentation. Case of acute APMPPE/AMIC analysed by ICGA & FA & BL-FAF. This multimodal imaging shows that the initial event is choriocapillaris non-perfusion clearly shown by extensive areas of ICGA hypofluorescence (middle two frames) while retinal (FA) involvement is still limited. BL-FAF hyperautofluorescence is still limited as choriocapillaris non-perfusion induced ischaemia did not alter the outer retina yet, except in a few areas along the superior temporal arcades.

For precise evaluation of areas of choriocapillaris non-perfusion and their follow-up, ICGA is by far the most valuable modality, revealing discrete to large areas of scattered or confluent hypofluorescent dark areas present from early to late angiographic frames [22,23,24] (Figure 4a,b and Figure 5).

**Figure 6:**
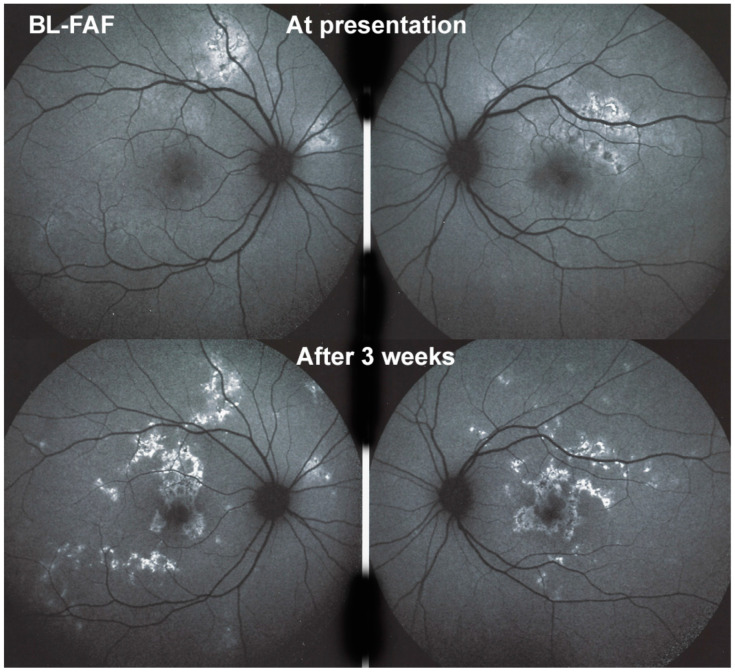
APMPPE/AMIC; BL-FAF at presentation and in the subacute phase. At presentation (top two frames) there is limited hyperautofluorescence as there is mainly thickening of the outer retina due to choriocapillaris non-perfusion (see Figure 8) and limited areas (yet) of loss of photoreceptor outer segments (see Figure 7). During the subacute phase (bottom two frames) there are extended irregular areas of hyperfluorescence corresponding either to loss of photoreceptor outer segments allowing to see the hyperautofluorescent RPE lipofuscin or RPE cell damage with an accumulation of cellular fluorophore debris or both.

**Figure 7:**
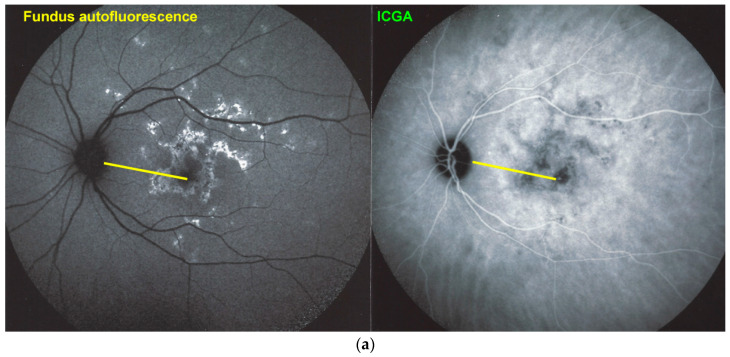

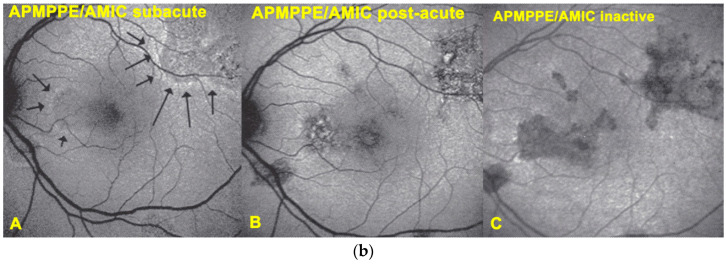
(**a**) APMPPE/AMIC; BL-FAF patterns in subacute stage (same patient as Figure 5 and Figure 6). Severe case after 3 weeks of evolution. BL-FAF shows central hypoautofluorescence corresponding to atrophy with loss of RPE, co-localising with dark ICGA hypofluorescence (yellow callipers), surrounded by hyperautofluorescence corresponding to non-perfusion with intact RPE and loss of photoreceptor segments and/or damaged RPE cells with accumulation of fluorophore debris. (Same patient as Figure 5 and Figure 6). (**b**) APMPPE/AMIC; BL-FAF evolution during stages of the disease. In the subacute stage (**A**) hyperautofluorescence is mainly present at the border of non-perfused areas (black arrows) but less so in the middle of diseased areas, indicating mostly non-functioning RPE cells while at the rim RPE cells are still metabolically active and lipofuscins can be seen through areas that have lost photoreceptor outer segments. At a later stage (**B**), hyperautofluorescent rims around the affected have disappeared because of healed RPE cells and reconstitution of the photoreceptor outer segment line. In the inactive healed phase (**C**) the chorioretinal scars where RPE cells have disappeared are hypoautofluorescent (Reprinted from Gupta A, Gupta V, Herbort CP, Khairallah M, editors. Uveitis, text and imaging. New Delhi: Jaypee Brothers Medical Publishers; 2009:328).

Blue light fundus autofluorescence (BL-FAF) shows both hyperautofluorescence and hypoautofluorescence depending on the stage of evolution of the disease and is sometimes difficult to interpret. In the very acute stage, there are still limited areas of hyperautofluorescence following the loss of photoreceptor outer segments due to non-perfusion which has caused ischaemia without chorioretinal atrophy. In the subacute stage, there is an alternation of hyperautofluorescence either due to loss of photoreceptor outer segments or damage of RPE cells with accumulation of fluorophore debris, or hypoautofluorescence due to chorioretinal atrophy with loss of RPE [5,7,25] (Figure 6 and Figure 7a,b). In progressing lesions hypoautofluorescence is central (atrophy) and hyperautofluorescence is located in the periphery of lesions where ischaemia causes photoreceptor outer segment loss with still conserved RPE and/or RPE cells with accumulation of cellular fluorophore debris (Figure 7a,b).

The evolution of BL-FAF findings during the different disease stages is illustrated in Figure 7b.

**Figure 8:**
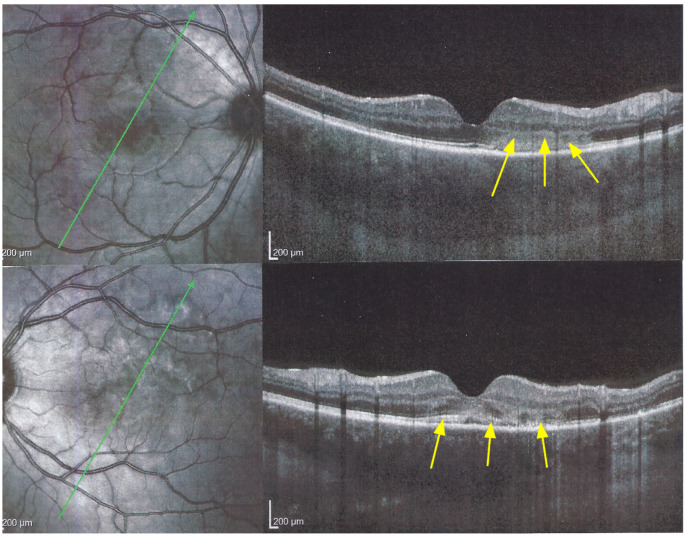
APMPPE/AMIC; SD-OCT in initial-acute phase OD (top) and OS (bottom) (same patient as Figure 5, Figure 6 and Figure 7a). In this initial phase, SD-OCT shows areas of hyperreflective changes visible in the outer plexiform layer and adjacent outer nuclear layer (arrows) but no loss of photoreceptor outer segments (see Figure 5).

**Figure 9:**
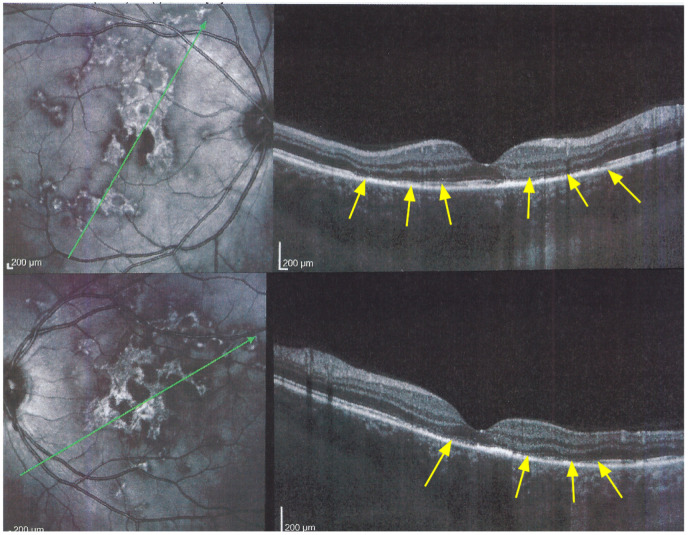
APMPPE/AMIC; SD-OCT in subacute phase OD (top) and OS (bottom) (same patient as Figure 5, Figure 6, Figure 7a and Figure 8). In the subacute phase, SD-OCT shows extended areas of loss of photoreceptor outer segments (arrows) and thickened RPE, hyperautofluorescent on BL-FAF (see Figure 5).

The chorioretinal findings by spectral domain optical coherence tomography (SD-OCT) depend on the degree of involvement and the stage of the disease [5,7,25]. In early-acute disease ischaemia, induced by choriocapillaris non-perfusion, causes thickening of the outer retina including the IS/OS line and beyond (Figure 8). In later stages, SD-OCT can show either simple loss of photoreceptor outer segments, thickened RPE or atrophy with loss of RPE (Figure 9).

**Figure 10:**
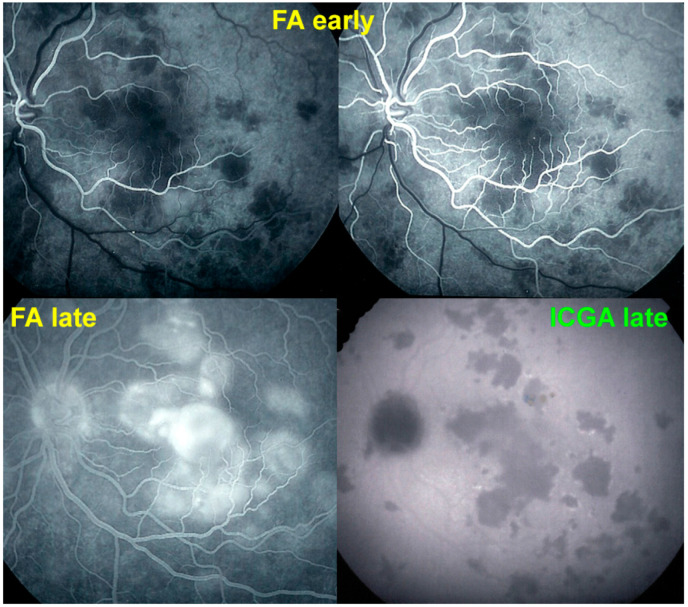
Late FA pooling in a severe case of APMPPE/AMIC. In severe cases of APMPPE/AMIC, FA shows choriocapillaris non-perfusion in early angiographic frames (top two pictures—FA early) followed by areas of abundant retinal pooling in the late angiographic phase (bottom left picture—FA late). Traditionally this phenomenon is explained by the very hypothetical alleged change of polarity of the RPE and fluid movement from the choroid to the retina. However, the choriocapillaris areas under the retinal pooling are non-perfused (bottom right picture—ICGA late). Therefore, a more probable origin of the fluid is an exudation from retinal vessels in response to severe outer retinal ischemia.

Interestingly, in some cases, the degree and extension of outer retina ischaemia in early-acute disease are such that, similar to diabetic retinal ischaemia, it causes reactionary dilatation and exudation of inner retinal vessels causing pooling visible on FA and visible as serous detachments on SD-OCT [25] (Figure 10).

**Figure 11:**
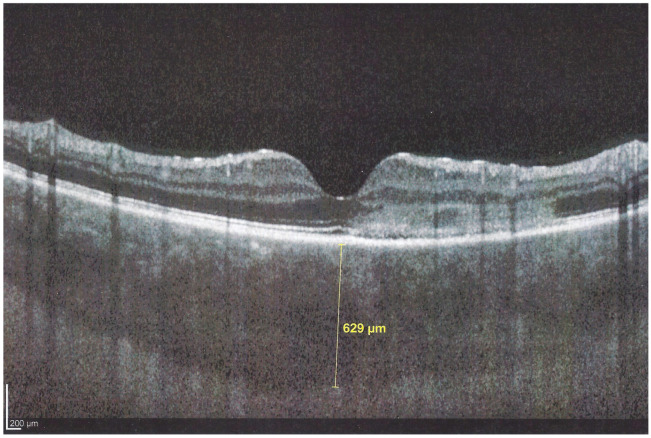
APMPPE/AMIC; EDI-OCT in the acute phase (same patient as Figure 5, Figure 6, Figure 7a, Figure 8 and Figure 9). In the acute phase, EDI-OCT shows an important thickening of the whole choroid with the calliper indicating a thickness of 629 µm.

**Figure 12:**
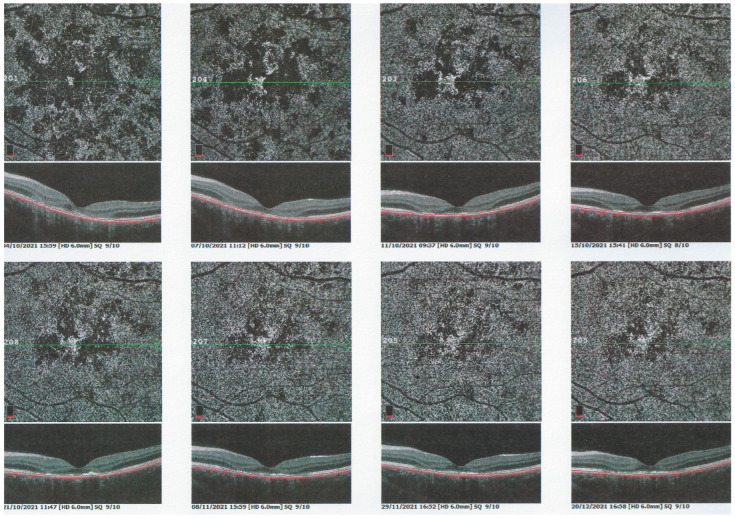
APMPPE/AMIC; OCT-angiography (OCT-A). OCT-A shows the choriocapillary drop-out at presentation (top left frame) and its progressive decrease up to 3 months after the onset of the disease (bottom right frame).

In addition to choriocapillaris non-perfusion, in most acute cases, the entire choroid is thickened, which can be shown by enhanced depth imaging OCT (EDI-OCT) (Figure 11). In complement to ICGA, OCT angiography (OCT-A) shows areas of choriocapillary drop-out that correlate with the areas of ICGA hypofluorescence the analysed area being however, limited to part of the posterior pole and less precise than ICGA [5,7,25] (Figure 12).

The prognosis for APMPPE cases varies from one case to the other and depends on the location and severity of involvement. In the literature, it is usually described as a self-limited condition. As indicated here above, there are reports of cases with extensive lesions and marked visual impairment needing systemic corticosteroid treatment. Additional immunosuppression does not seem to be necessary in most cases [8,9,10,11]. Cerebral vasculitis is a rare complication or co-morbidity that has to be suspected when neurological symptoms are noted possibly confirmed by a positive cerebral magnetic resonance imaging angiography (angio-MRI) [26,27]. Systemic vasculitis associated with APMPPE has also been reported [28]. Most numerous complications include chorioretinal scars and, as is always possible with chorioretinal scars, the development of CNVs needing intravitreal anti-VEGF therapy.

The aim of this study was to retrospectively analyse a relatively large series of APMPPE/AMIC patients seen in our centre, determine their clinical and imaging characteristics, and establish the proportion of patients with a more pronounced disease where systemic prednisone therapy was deemed necessary and compare these findings to the literature. In patients that were followed in our centre, the evolution of cases and outcomes were documented.

## 2. Patients and Methods

This retrospective case series was performed in the Centre for Ophthalmic Specialised Care (COS), Lausanne, Switzerland. Patients diagnosed from 2000 to 2021 with APMPPE/AMIC were included. Patients having a positive QuantiFERON test and/or positive VDRL-TPHA test were excluded. Imaging analysis included spectral domain optical coherence tomography (SD-OCT) and enhanced depth imaging OCT (EDI-OCT) (Heidelberg Engineering GmbH, Heidelberg, Germany), OCT angiography (OCT-A) (AngioVue^®^, Optovue, Fremont, CA, USA) Fluorescein and Indocyanine angiography (FA, ICGA) (Heidelberg Engineering GmbH, Heidelberg, Germany). Best corrected visual acuity (BCVA) and routine ocular examination, as well as laser flare photometry (LFP), were performed at presentation and during the follow-up of patients. The presence or not of a prodromal febrile and/or viral episode or other systemic symptoms was noted. Eyes were divided into 2 groups, depending on the localisation of the lesions by ICGA and/or SD-OCT. The lesions were qualified as foveal when the hypofluorescent areas included the fovea on ICGA and/or when alterations of the outer retina and/or RPE were noted in the fovea by SD-OCT. The lesions were characterised as perifoveal or parafoveal when hypofluorescent areas were spotted outside the fovea by ICGA and/or when the outer retina was untouched in the fovea by SD-OCT.

## 3. Results

### 3.1. Demographics

Nineteen out of 1664 new patients (35 eyes) (1.14%) were diagnosed with APMPPE/AMIC in the uveitis clinic of the Centre for Ophthalmic Specialised Care (COS) during the period from 2000 to 2021 and were included in our study. 13 (68%) were male and 6 (32%) were female. The mean age was 33.1 ± 9.2 years. 16 (84%) patients mentioned a viral prodromal episode, or other systemic symptoms and 3 (16%) did not mention any episode before the onset of the ocular symptoms.

### 3.2. Clinical Findings

The series was divided into 2 groups depending on whether lesions were foveal or extra-foveal. 15 of 38 eyes (38%) had a foveal location of lesions while 20/38 (52.6%) eyes had parafoveal or peri-foveal lesions and 3 eyes were normal in the 3 unilateral cases (15%).

The mean BCVA for the whole series at presentation was 0.83 ± 0.24. The mean flare at presentation was 10.52 ± 7.7, indicating minimal anterior segment involvement. One patient had bilateral non-granulomatous uveitis with synechiae. 3 eyes presented choroidal neovascular membranes that were treated by 4 intravitreal injections of anti-VEGF agents.

**Figure 13:**
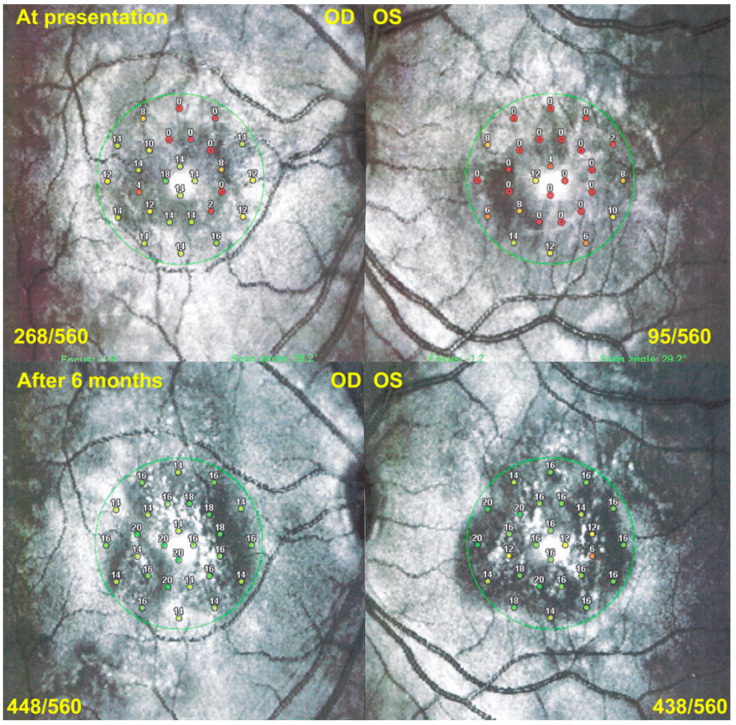
APMPPE/AMIC; Microperimetry (MP). MP of a case of APMPPE/AMIC at presentation (top two pictures) showing a decrease of retinal sensitivity to 268/550 (OD) and 95/560, recovering to 448/560 (OD) and 438/560 (OS) after 6 months (bottom two pictures).

Visual field (VF) showed central and/or paracentral scotomas with a mean MD at a presentation of 5 ± 3.6 db. However, microperimetry, when available, showed a much more precise topography of the decreased retinal sensitivity and a numerical measurement at presentation and on follow-up (Figure 13).

### 3.3. Imaging Findings

The most important and constant imaging modality was **ICGA** which showed early to late hypofluorescent dots/areas scattered or confluent taking a geographic pattern. This finding was present in absolutely all 35/35 eyes and is the hallmark of the disease. Lesions were hypofluorescent from early to late angiographic frames. (Figure 4a,b, Figure 5, Figure 7 and Figure 10).

**Figure 14:**
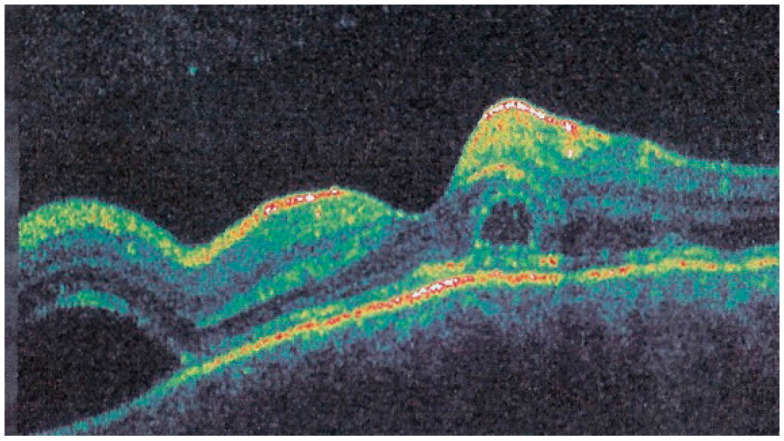
APMPPE/AMIC SD-OCT; Subretinal fluid producing serous retinal detachment (SRDs) and intra-photoreceptor layer separation, in a hyperacute case.

**Figure 15:**
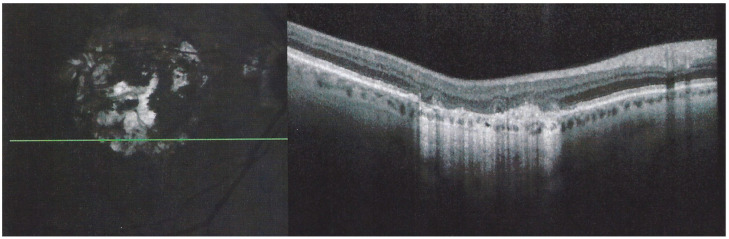
APMPPE/AMIC SD-OCT; Chorioretinal atrophy in the late stage.

**SD-OCT** findings included thickened hyperreflective areas of the outer retina in the very early disease phase (10/35 eyes) (Figure 8 and Figure 11), photoreceptor outer segment loss and/or ellipsoid zone disruption-RPE alterations (22/35 eyes) (Figure 9), subretinal fluid (serous retinal detachment—SRD) (4/35 eyes), (Figure 14) and atrophy (3/35 eyes) in the late stage of the disease (Figure 15).

**FA** consistently showed early hypofluorescence with late hyperfluorescence in 19/35 eyes, (Figure 10) early hypofluorescence with late iso-fluorescence in 4/35 eyes, and disc hyperfluorescence in 6/35 eyes while in 2 eyes there was early to late hypofluorescence due to extended chorioretinal atrophy including the RPE.

In hyperacute cases, there was late subretinal pooling corresponding to the SDR on SD-OCT found in 4/35 eyes (Figure 10).

**BL-FAF:** 3 pathological eyes had normal FAF, while hyperautofluorescence mixed with hypoautofluorescence was detected in 5/23 eyes; (Figure 7) hyperautofluorescence alone was detected in 12/23 eyes and hypoautofluorescence in 3/23 eyes. There were no FAF data available in 6 patients.

**Figure 16:**
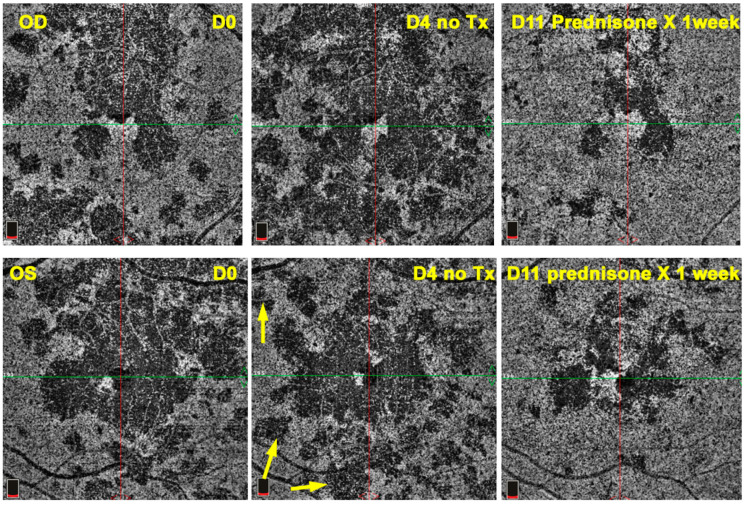
APMPPE/AMIC OCT-A; Case of hyperacute APMPPE/AMIC with numerous hypo-perfused areas at presentation (D0). Prednisone treatment was withheld until negative syphilis serology was available with the progression of non-perfused areas (D4 yellow arrows). One week after the introduction of systemic prednisone therapy (D11) lesions had regressed substantially.

**OCT-A** showed patchy choriocapillaris drop-out in acute phases and absence of signal in atrophic lesions, but it was not available for 12 patients (Figure 16).

### 3.4. Localisation of Lesions and Visual Outcomes

While BCVA for the whole series at presentation amounted to 0.83 ± 0.24, in the 15 eyes of 12 patients that had foveal lesions, BCVA at presentation was 0.58 ± 0.28, increasing to 0.97 ± 0.13 at final follow-up, a difference that was statistically significant (*p* = 0.0028). For the 20 eyes of 15 patients that presented with peri/parafoveal localisation, BCVA at presentation was 0.94 ± 0.18 while at the last follow-up it was 1.18 ± 0.10, also statistically significant (*p* = 0.0039).

### 3.5. Corticosteroid/Immunosuppressive Treatment and Visual Outcome

13/19(68%). patients had received systemic prednisone therapy and 2 patients (11%) had received additionally at least one immunosuppressive agent. 4 (21%) patients had received no treatment. No information was available for 2 patients (11%). Treatment was either already given by the referring ophthalmologist or by us. 11 out of 12 patients with foveal involvement of at least 1 eye had received systemic prednisone treatment (3 patients with bilateral foveal lesions, 9 patients with unilateral foveal lesions). One referred patient with bilateral foveal involvement had not received any treatment and was seen in our centre with severe bilateral foveal atrophy as a consequence and visual acuities of 0.1 bilaterally. Among the patients with peri/parafoveal localization, 12 eyes (10 patients) had received treatment, 5 eyes (3 patients) had not received treatment, and for 3 eyes (2 patients), no information was available. 3/35 eyes (8%) had received intraocular anti-VEGF injections because of choroidal neovascularisation.

25/32(78%). eyes had received oral corticosteroid/immunosuppressive therapy of which follow-up data were available for 19 eyes. BCVA at presentation was 0.74 ± 0.28 versus 1.06 ± 0.15 at the last follow-up. (*p* < 0.0001).

We have data of follow-up from only 5 eyes that received no treatment. BCVA at presentation was 0.64 ± 0.22 versus 0.66 ± 0.4 at last follow, not significantly increased (*p* = 0.17). This indicates that the eyes were probably doing better functionally when treated.

## 4. Selected Case Reports

### 4.1. Case 1: Typical Presentation

**Figure 17:**
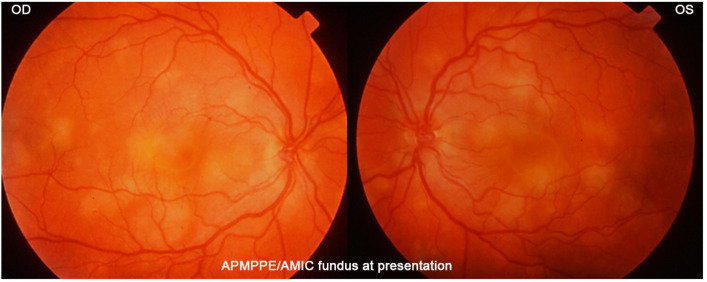
APMPPE/AMIC: Typical case of a 27-year-old patient showing the characteristic yellow-white placoid fundus lesions.

**Figure 18:**
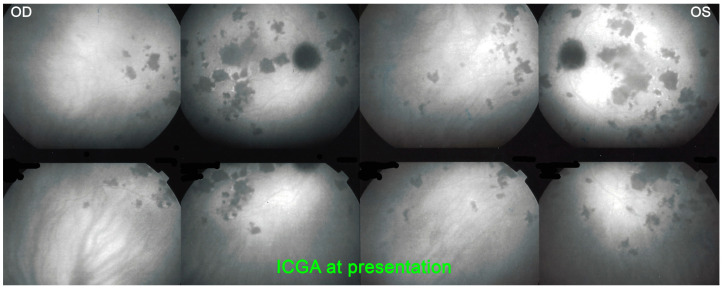
APMPPE/AMIC, ICGA at presentation: Characteristic bilateral hypofluorescent areas representing choriocapillaris non-perfusion.

**Figure 19:**
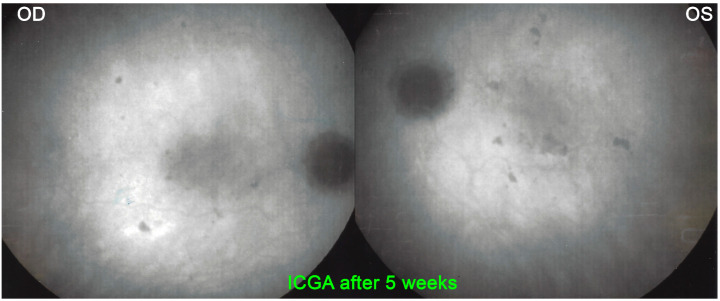
APMPPE/AMIC, ICGA after 5 weeks: Resolution of most of the hypofluorescent areas after systemic prednisone treatment.

A 27-year-old student noticed a decrease in visual acuity in both eyes while preparing for his exams and sought consultation in an emergency. He said he was in a suboptimal general condition in the past 3 weeks with tiredness, loss of appetite, weight loss and joint pain. BCVA was 0.7 OD and 0.5 OS. There were cells (1+) in the anterior chamber and a slightly elevated flare measured by laser flare photometry amounting to 11.5 ph/ms OD and 9.9 ph/ms OS, as well as rare vitreous cells. Fundus examination showed bilateral yellow-white placoid lesions in the posterior pole and the midperiphery (Figure 17). ICGA showed the characteristic geographic partially confluent hypofluorescent areas in both eyes (Figure 18). FA showed areas of choriocapillaris non-perfusion in the posterior pole in the early frames (Figure 10). In the later frames, it showed areas of subretinal pooling co-localising with the areas of ICGA hypofluorescence (Figure 10). Treatment of prednisolone 1% drops 5X daily was started. However, the patient returned the same evening because of a further drop in vision. BCVA had fallen to 0.2 bilaterally and the patient was very anxious as he was preparing for his university tests. While waiting for the results of syphilis serology, the patient was only treated 48 h after the first presentation with 50 mg of prednisone leading to the recovery of 1.0 VA in both eyes 2 weeks later. Prednisone was discontinued 4 weeks later and a repeat angiography 5 weeks after the onset of the disease showed the disappearance of most of the hypofluorescent areas on ICGA and a normal FA (Figure 19). It is not possible to know whether the recovery would have occurred without corticosteroid therapy. However, in the case of functional and/or morphological/imaging deterioration, refraining from systemic corticosteroid therapy is a difficult position to hold despite the allegedly self-limited character of the disease. Taking into account the low risk/potential benefit ratio, the intervention will never be blamable for this crucial part of the retina.

### 4.2. Case 2: Deleterious Evolution in an Untreated Case

**Figure 20:**
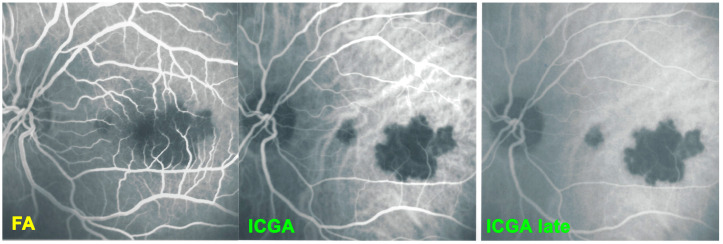
APMPPE/AMIC at first presentation elsewhere. FA early frame (**left**) shows two areas of non-perfusion, which are better delineated by the ICGA intermediate phase (**middle frame**) and late phase (**right frame**).

A 55-year-old man presented a febrile “viral” episode 3 weeks after travelling to Indochina followed by a decrease of vision in his left eye with photopsia. The diagnosis of APMPPE was made elsewhere and it was decided not to introduce systemic treatment “as the disease is self-limited”. FA/ICGA performed was compatible with APMPPE/AMIC (Figure 20).

**Figure 21:**
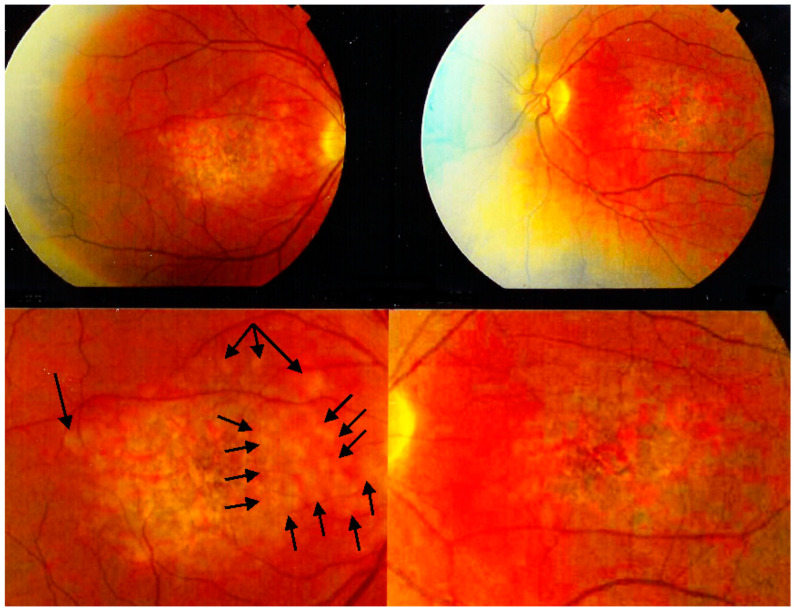
APMPPE/AMIC case not having received prednisone therapy with an evolution towards macular atrophy ODS: This patient presented with macular atrophy OS following involvement first of the left eye and placoid lesions in the right fundus still partially active (black arrows).

**Figure 22:**
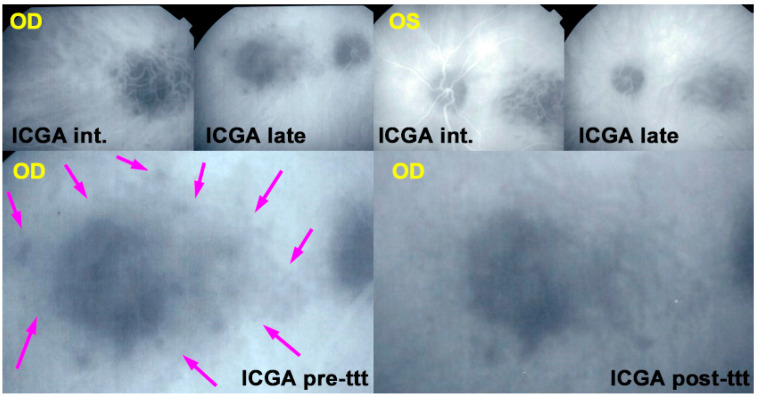
APMPPE/AMIC; ICGA in a case that did not receive prednisone therapy with an evolution towards macular atrophy ODS: top frames show ICGA findings in intermediate and late phases in both eyes at presentation 3 months after the onset of disease. The bottom left frame shows active perimacular lesions less hypofluorescent than the centre (crimson arrows) that responded to prednisone therapy with regression of perimacular lesions after treatment but the persistence of centromacular atrophy (bottom right frame).

However, the vision continued to decrease OS and when the same symptomatology occurred on the right side, the patient decided to consult another hospital where the same diagnosis of APMPPE was posed, acute in OD and subacute in OS and again no treatment was introduced because of the allegedly self-limited character of APMPPE/AMIC. When vision continued to decrease the patient consulted our centre. At presentation, BCVA was 0.05 OD and 0.15 OS. Fundus examination showed a cicatricial macula on the left and confluent atrophic plaques on the right with some foci still active (Figure 21). ICGA showed macular hypofluorescence due to macular atrophy on the left and central hypofluorescence due to atrophy on the right surrounded by less hypofluorescent areas indicating still active lesions (Figure 22). SD-OCT and FAF were not available at the time.

Therefore, treatment with 60 mg daily prednisone was given allowing to reverse the peripheral lesions OD (Figure 22) with an increase of BCVA to 0.15. No effect was obtained on the left eye that had been involved first where the macula was completely atrophic. In contrast to the first case, this patient did not receive treatment with a deleterious evolution, indicating that the evolution of the disease cannot be predicted and in case of doubt a treatment should be recommended with a potential low risk/benefit ratio.

### 4.3. Case 3: Typical Case with Multimodal Imaging

**Figure 23:**
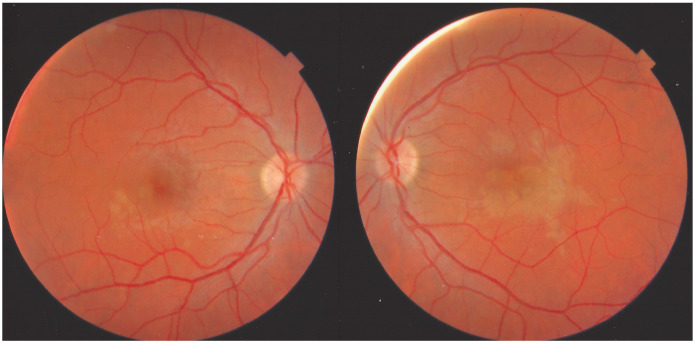
APMPPE/AMIC case at a subacute stage having received prednisone therapy for 10 days. Faint placoid lesions still visible OS and faint macular discolouration OD.

This 35-year-old man was seen in an emergency centre abroad for a bilateral decrease of vision associated with a febrile flu-like condition with muscle pain. Prednisone at the dosage of 50 mg had been started. We saw the patient 10 days later while he was still symptomatic in his left eye. BCVA was 1.0 OD and 0.5 OS. There was a slight subclinical anterior chamber inflammation measured with laser flare of 5.5 ph/ms OD and 11.3 ph/ms OS, but no vitritis. Fundus examination showed faint placoid lesions OS in the posterior pole and very faint discolouration of the macula OD (Figure 23).

**Figure 24:**
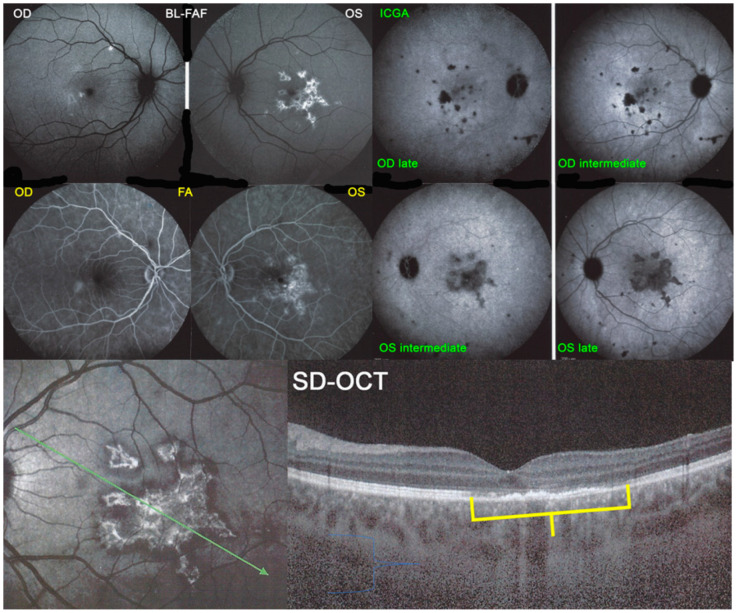
APMPPE/AMIC; BL-FAF. ICGA, FA & SD-OCT findings in a case in subacute stage having received prednisone therapy for 10 days: BL-FAF (top left two frames) shows areas of hyperautofluorescence (OS > OD) corresponding to the areas of hypofluorescence (choriocapillaris non-perfusion) on ICGA (top right quartet of frames). FA (middle left two frames) shows areas of staining (OS) most probably produced by exudation of retinal vessels in response to outer retinal ischaemia. SD-OCT (bottom two pictures) shows loss and disorganisation of photoreceptor outer segments, disrupted RPE layer with overlying hyperreflective focal deposits in correspondence to hyperautofluorescence.

**Figure 25:**
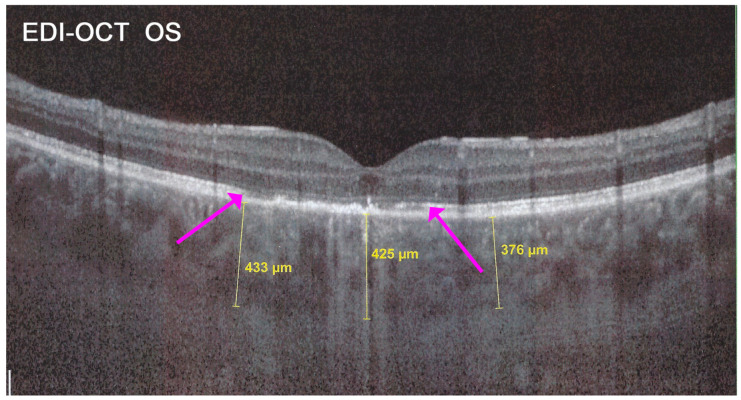
APMPPE/AMIC; EDI-OCT (same case as Figure 21 and Figure 22). Besides the loss of photoreceptor outer segments (marked by crimson arrows), the whole choroid is thickened, with callipers showing values between 376 µm and 433 µm.

The exact extensions of bilateral lesions were clearly delineated on ICGA showing scattered small hypofluorescent areas of choriocapillaris non-perfusion OD and more confluent hypofluorescent lesions OS (Figure 24), corresponding to areas of hyperautofluorescence on BL-FAF and FA hyperfluorescence (Figure 24). SD-OCT showed loss of photoreceptor outer segments and irregularity of the IS/OS line due to ischaemia following choriocapillaris non-perfusion (Figure 24). FA hyperfluorescence is explained by compensatory retinal exudation with limited pooling as the patient was already under corticosteroid therapy. Besides choriocapillaris non-perfusion, the whole choroidal thickness was increased (Figure 25).

**Figure 26:**
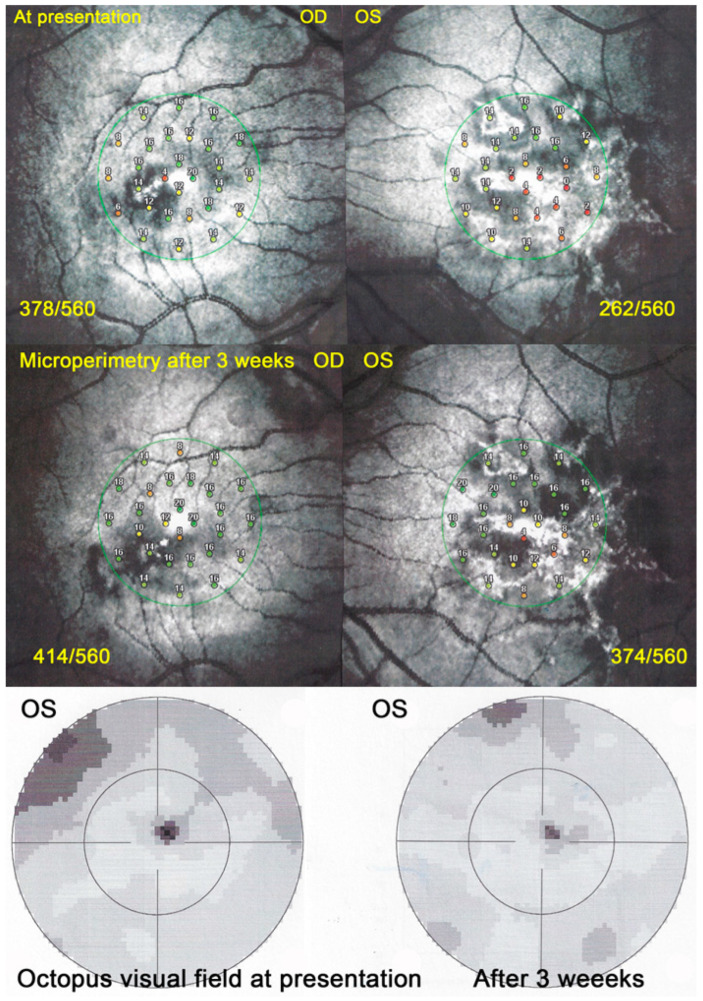
APMPPE/AMIC; Microperimetry and visual field evolution (same patient as Figure 21, Figure 22 and Figure 23): Microperimetry is closely and numerically monitoring the evolution of retinal sensitivity from 373/560 to 414/560 OD and from 262/560 to 374/560 OS and is much more precise than visual field testing (bottom 2 images).

Functional follow-up was best obtained by microperimetry, more sensitive than visual field testing and useful to monitor deleterious evolution (Figure 26). After 3 weeks the different imaging signs partially regressed in parallel with microperimetric improvement of retinal sensitivity from 378/560 to 414/560 (OD) and from 262/560 to 374/560.

## 5. Discussion

We report on 19 APMPPE/AMIC cases seen over a period of 22 years at the COS uveitis clinic, being among the largest series published when excluding meta-analysis series. This represented a percentage of 1.14% of all new patients seen during this period and corresponding to approximately 1 patient per year. Although the clinical presentation is usually characteristic, there is a variability of presentations from discrete to very severe cases which are reflected in our series. The evolution usually described in textbooks, series and case reports is that of a self-limited disease not necessarily needing systemic corticosteroid treatment. Therefore, astonishingly, in more than two-thirds of our patients, systemic corticosteroid therapy with or without additional non-steroidal immunosuppression was deemed necessary. Indeed, one non-treated referred patient lost both maculae to atrophy. We found a higher than reported rate of prodromal systemic illnesses including symptoms of viral disease or other amounting to close to 85%. Men were more frequent in our series than generally reported, representing a little over two-thirds; although as reported in other series, there does not seem to be a significant gender difference in APMPPE/AMIC [29]. Due to the lack of predictability of the evolution of the disease, close monitoring of non-treated patients should be the standard of management in order to intervene promptly with corticosteroid treatment in case of deleterious evolution. Close monitoring has become easier with the multimodal imaging modalities at our disposal nowadays. The gold standard to establish and closely follow lesions is without contest ICGA. The latter is also the modality that is able to show early lesions not yet demonstrated by the other modalities, indicating that the “primum movens” is indeed choriocapillaris non-perfusion at the origin of all other imaging signs. OCT-A represented a precise and non-invasive method to follow non-perfusion areas, able to show progression in non-treated cases and subsequent regression after the introduction of prednisone therapy. BL-FAF is more difficult to interpret in APMPPE/AMIC when compared to MEWDS where it represents almost exclusively loss of IS/OS line and can therefore be used for the follow-up. In APMPPE/AMIC BL-FAF shows both losses of photoreceptor outer segments together with accumulation of fluorophore debris in metabolically damaged RPE cells in more hardly hit areas, producing hyperautofluorescence of different patterns and proportions. When RPE cells are lost altogether hypoautofluorescence is noted. Initial SD-OCT signs, in the acute phase of the disease, showed thickening of the outer retina which became hyperreflective and, later, loss of IS/OS line of photoreceptor outer segments with a focal thickness of the RPE in more hardly hit areas. The most characteristic FA sign in severe disease was late pooling explained by the exudation from retinal vessels in reaction to outer retina ischaemia, sometimes producing SRD in SD-OCT.

Outcomes were favourable without treatment in eyes with extrafoveal lesions. Visual outcome in eyes with and without foveal involvement was better in eyes treated with corticosteroids when compared to non-treated eyes, with a good visual acuity and good recovery of visual fields. This was in contrast to the one non-treated patient with severe lesions the location of which included the fovea leading to a deleterious evolution. Despite the publication of cases and a series reporting favourable outcomes without treatment [30], abstention may be harmful. In case of potentially dangerous location of lesions and view of the unpredictability of the disease together with the numerous publications indicating that the notion of the self-limited character of the disease is incorrect [9,10,11,31,32], a short course of corticosteroids represents a limited risk. Therefore, the decision to introduce prednisone therapy should be taken liberally, unless lesions are far from the central macula and not potentially vision-threatening.

Choroidal neovascular membranes occurred in 3 of 35 eyes but no central nervous system complications such as cerebral vasculitis were noted in our collective.

Many articles in the literature report cases resembling APMPPE/AMIC; however, they are lacking the classical criteria. These cases should better be termed as APMPPE/AMIC-like cases or undefined choriocapillaritis cases.

**Scheme 1:**
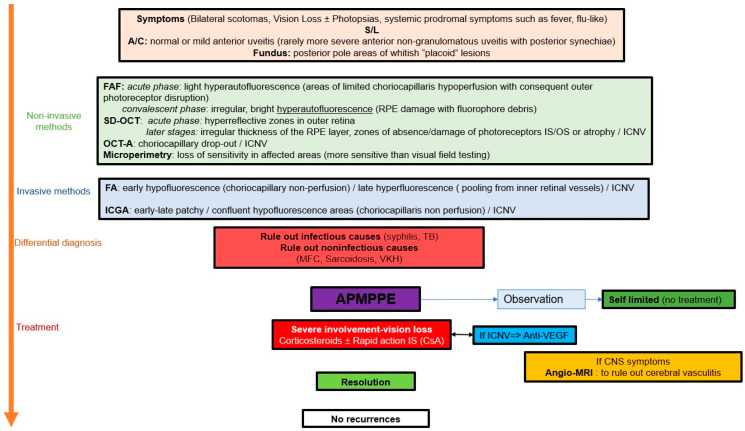
APMPPE/AMIC: decision tree in clinical practice.

The key to responsible management of APMPPE/AMIC is the establishment of a clear delineation of lesions at presentation obtained thanks to ICGA, followed by precise and close monitoring of lesions which is very much helped by non-invasive imaging modalities at our disposal such as SD-OCT, BL-FAF and OCT-A, as well as functional investigations with a high grade of precision such as microperimetry (Scheme 1).

## Data Availability

Please refer to the corresponding author.

## References

[1] Gass J.D.M. (1968). Acute Posterior Multifocal Placoid Pigment Epitheliopathy. Arch. Ophthalmol..

[2] Deutman A.F., Oosterhuis J.A., Boen-Tan T.N., Aan de Kerk A.L. (1972). Acute posterior multifocal placoid pigment epitheliopathy. Pigment epitheliopathy or choriocapillaritis. Br. J. Ophthalmol..

[3] Deutman A.F., Lion F. (1977). Choriocapillaris non-perfusion in acute multifocal placoid pigment epitheliopathy. Am. J. Ophthalmol..

[4] Howe L.J., Woon H., Graham E.M., Fitzke F., Bhandari A., Marshall J. (1995). Choroidal hypoperfusion in acute posterior multifocal placoid pigment epitheliopathy. An indocyanine green angiography study. Ophthalmology.

[5] Herbort C.P., Mantovani A., Tugal-Tutkun I., Papasavvas I. (2021). Classification of Non-Infectious and/or Immune Mediated Choroiditis: A Brief Overview of the Essentials. Diagnostics.

[6] Herbort C.P., Neri P., Papasavvas I. (2021). Clinicopathology of non-infectious choroiditis: Evolution of its appraisal during the last 2–3 decades from “white dot syndromes” to precise classification. J. Ophthalmic Inflamm. Infect..

[7] Papasavvas I., Neri P., Mantovani A., Herbort C.P. (2022). Idiopathic multifocal choroiditis (MFC): Aggressive and prolonged therapy with multiple immunosuppressive agents is needed to halt the progression of active disease. An offbeat review and a case series. J. Ophthalmic Inflamm. Infect..

[8] Daniele S., Daniele C., Orcidi F., Tavano A. (1998). Progression of choroidal atrophy in acute posterior multifocal placoid pigment epitheliopathy. Ophthalmologica.

[9] Sulewski M.E., Kolomeyer A.M., Saran B.R., Brucker A.J. (2021). A 15-year-old boy with protracted vision loss from acute posterior multifocal placoid pigment epitheliopathy. Retin. Cases Brief Rep..

[10] El-Markaby H.S., Mohammed T.H., El-Raggal T.M. (2012). Acute posterior multifocal placoid pigment epitheliopathy: Role of TNF blocker in severe cases. Retina.

[11] Pagliarini S., Piguet B., Ffytche T.J., Bird A.C. (1995). Foveal involvement and lack of visual recovery in APMPPE associated with uncommon features. Eye.

[12] Testi I., Vermeirsch S., Pavesio C. (2021). Acute posterior multifocal placoid pigment epitheliopathy (APMPPE). J. Ophthalmic Inflamm. Infect..

[13] Yang C.-S., Hsieh M.-H., Su H.-I., Kuo Y.-S. (2019). Multiple Evanescent White Dot Syndrome Following Acute Epstein-Barr Virus Infection. Ocul. Immunol. Inflamm..

[14] Kraemer L.S., Montgomery J.R., Baker K.M., Colyer M.H. (2022). Acute posterior multifocal placoid pigment epitheliopathy after immunization with multiple vaccines. Retin. Cases Brief Rep..

[15] Branisteanu D., Bilha A. (2015). Acute posterior multifocal placoid pigment epitheliopathy following influenza vaccination. Rom. J. Ophthalmol..

[16] Atas F., Kaya M., Saatci A.O. (2021). Acute Multifocal Placoid Pigment Epitheliopathy-like Presentation following the First Dose of BNT162B2 COVID-19 Vaccination. Ocul. Immunol. Inflamm..

[17] Jyotirmay B., Jafferji S.S., Sudharshan S., Kalpana B. (2010). Clinical profile, treatment, and visual outcome of ampiginous choroiditis. Ocul. Immunol. Inflamm..

[18] Borruat F.-X., Piguet B., Herbort C.P. (1998). Acute posterior multifocal placoid pigment epitheliopathy following mumps. Ocul. Immunol. Inflamm..

[19] Herbort C.P., Papasavvas I., Mantovani A. (2020). Choriocapillaris involvement in Acute Syphilis Posterior Placoid Chorioretinitis is responsible for functional impairment and points towards an immunologic mechanism: A comprehensive clinicopathological approach. J. Curr. Ophthalmol..

[20] Papasavvas I., Jeannin B., Herbort C.P. (2022). Tuberculosis-related serpiginous choroiditis: Aggressive therapy with dual concomitant combination of multiple anti-tubercular and multiple immunosuppressive agents is needed to halt the progression of the disease. J. Ophthalmic Inflamm. Infect..

[21] Herbort C.P., Tugal-Tutkun I., Mantovani A., Neri P., Khairallah M., Papasavvas I. (2020). Advances and potential new developments in imaging techniques for posterior uveitis Part 2: Invasive imaging methods. Eye.

[22] Tugal-Tutkun I., Herbort C.P., Mantovani A., Neri P., Khairallah M. (2021). Advances and potential new developments in imaging techniques for posterior uveitis. Part 1: Noninvasive imaging methods. Eye.

[23] Dhaliwal R.S., Maguire A.M., Flower R.W., Arribas N.P. (1993). Acute posterior multifocal placoid pigment epitheliopathy. An indocyanine green angiographic study. Retina.

[24] Cimino L., Auer C., Herbort C.P. (2000). Sensitivity of indocyanine green angiography for the follow-up of active inflammatory choriocapillaropathies. Ocul. Immunol. Inflamm..

[25] Papasavvas I., Herbort C.P. (2022). Diagnosis and Treatment of Primary Inflammatory Choriocapillaropathies (PICCPs): A Comprehensive Overview. Medicina.

[26] Çomu S., Verstraeten T., Rinkoff J.S., Busis N.A. (1996). Neurological manifestations of acute posterior multifocal placoid pigment epitheliopathy. Stroke.

[27] Tsang B.K.-T., Chauhan D.S., Haward R., Whiteman I., Frayne J., McLean C. (2014). Fatal ischemic stroke complicating acute multifocal placoid pigment epitheliopathy: Histopathological findings. J. Neuro-Ophthalmol..

[28] Hsu C.T., Harlan J.B., Goldberg M.F., Dunn J.P. (2003). Acute posterior multifocal placoid pigment epitheliopathy associated with a systemic necrotizing vasculitis. Retina.

[29] Faia L.J. (2014). Gender differences in birdshot chorioretinopathy and the white dot syndromes: Do they exist?. J. Ophthalmol..

[30] Xerri O., Salah S., Monnet D., Brézin A.P. (2018). Untreated Acute Posterior Multifocal Placoid Pigment Epitheliopathy (APMPPE): A case series. BMC Ophthalmol..

[31] Damato B.E., Nanjiani M., Foulds W.S. (1983). Acute posterior multifocal placoid pigment epitheliopathy. A follow up study. Trans. Ophthalmol. Soc. UK.

[32] Roberts T.V., Mitchell P. (1997). Acute posterior multifocal placoid pigment epitheliopathy: A long-term study. Aust. N. Z. J. Ophthalmol..

